# Connexin 36 Expression Regulates Neuronal Differentiation from Neural Progenitor Cells

**DOI:** 10.1371/journal.pone.0014746

**Published:** 2011-03-09

**Authors:** Elizabeth M. Hartfield, Federica Rinaldi, Colin P. Glover, Liang-Fong Wong, Maeve A. Caldwell, James B. Uney

**Affiliations:** 1 Henry Wellcome Laboratories for Integrative Neuroscience & Endocrinology, Laboratories for Integrated Neuroscience and Endocrinology, University of Bristol, Bristol, United Kingdom; 2 Neurosciences CEDD, GlaxoSmithKline, Harlow, United Kingdom; The University of Akron, United States of America

## Abstract

**Background:**

Gap junction communication has been shown in glial and neuronal cells and it is thought they mediate inter- and intra-cellular communication. Connexin 36 **(**Cx36) is expressed extensively in the developing brain, with levels peaking at P14 after which its levels fall and its expression becomes entirely neuronal. These and other data have led to the hypothesis that Cx36 may direct neuronal coupling and neurogenesis during development.

**Methodology/Principal Findings:**

To investigate Cx36 function we used a neurosphere model of neuronal cell development and developed lentiviral Cx36 knockdown and overexpression strategies. Cx36 knockdown was confirmed by western blotting, immunocytochemistry and functionally by fluorescence recovery after photobleaching (FRAP). We found that knockdown of Cx36 in neurosphere neuronal precursors significantly reduced neuronal coupling and the number of differentiated neurons. Correspondingly, the lentiviral mediated overexpression of Cx36 significantly increased the number of neurons derived from the transduced neurospheres. The number of oligodendrocytes was also significantly increased following transduction with Cx36 indicating they may support neuronal differentiation.

**Conclusions/Significance:**

Our data suggests that astrocytic and neuronal differentiation during development are governed by mechanisms that include the differential expression of Cx36.

## Introduction

Gap junction channels are formed when connexin (Cx) subunits situated in adjacent plasma membranes dock together. These transmembrane channels allow the passage of metabolites, ions and second messengers and nucleotides up to 1 kDa in size [1]. Undocked connexins, or hemichannels have also been identified and they provide a means to contact the extracellular environment. For example, they allow the passage of low molecular weight molecules into the cell [2], [3] and are gated by the cations Na^+^, K^+^ and Ca^2+^ [4], [5]. Gap junction communication has been shown in glial and neuronal cells and recently Cx43 was shown to negatively modulate neuronal differentiation [6]. Cx36 is also hypothesized to play a role in neuronal development, because its expression peaks in the inferior olive, cerebellum, striatum, hippocampus and cerebral cortex during the first 3 postnatal weeks, a period that coincides with extensive inter-neuronal coupling [7]. Importantly, during development Cx36 expression becomes restricted to neuronal cells, while Cx43 expression becomes restricted to astrocytes [8], [9]. Cx36 is expressed dynamically during murine embryonic development and it is switched on earlier than other Cxs. Expression is evident at E9.5 in the forebrain and expands into the midbrain as neurogenesis occurs. By E12.5 its expression pattern matches that of major morphogenetic boundaries within the brain and this elevated pattern of expression continues until P14. This bimodal pattern of neural expression correlates with two major periods of circuit formation and further indicates a role for Cx36 in the fine-tuning of neural development [8], [9].

Neural stem cells have been identified in both the developing and adult nervous systems [10]-[12]. These cells are self-renewing and can give rise to neurons, astrocytes and oligodendrocytes in the central nervous system (CNS). Functional gap junction proteins (including Cx43 and Cx36) have been identified in neural and embryonic stem cells and they are thought to play an important role in cell survival and differentiation [13]. Current data suggests that widespread Cx expression is required for synchronizing and fine-tuning developing populations of cells and their expression is both spatially and temporally regulated during mammalian CNS development [14]. In this study we investigated the role Cx36 plays during neuronal differentiation from neural stem cells and lentiviral over expression and knockdown strategies demonstrated that neural differentiation is positively influenced by Cx36.

## Materials and Methods

### Construction of Cx36 shRNA lentiviral vectors

**Figure 1:**
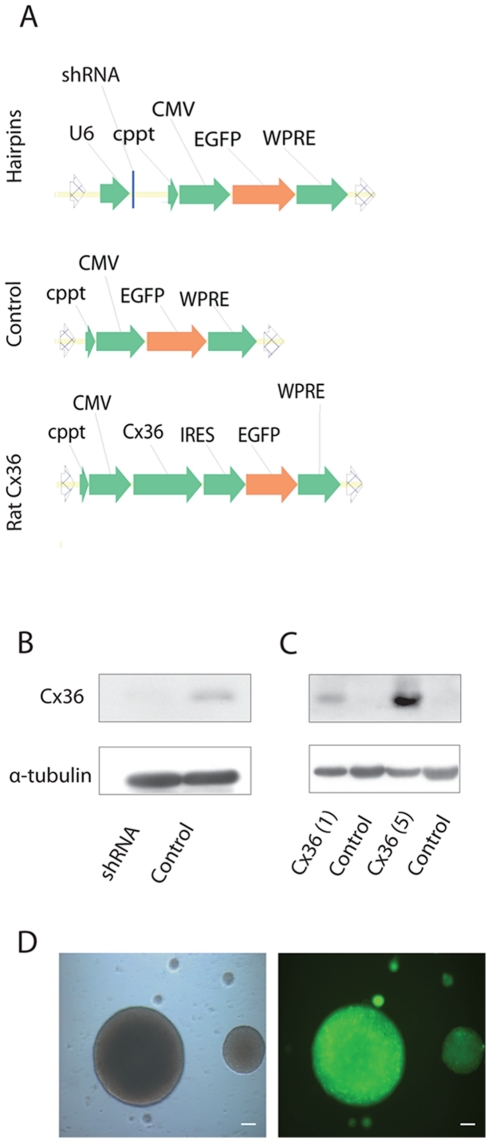
Validation of lentiviral expression cassettes. Lentiviral expression cassettes (A). Cx36 levels in neuronal cultures were assessed by western blotting following transduction with a lentivirus expressing an anti-Cx36 shRNA (B) and one overexpressing Cx36 (C). Rat neurosphere cultures transduced with a lentivirus expressing EGFP (D). Scale bar = 100 µm.

Rat (NCBI accession number Y16898) Cx36 shRNA (5′CAGATTGCTTAGAGGTTAA 3′) and scrambled control (5′GGCACATTAGGAACCATACAT 3′) sequence primers were designed and sub-cloned into pCR2.1-TOPO (Invitrogen) as previously described [15]. The resulting plasmid was digested with EcoRI and the U6.Cx36.shRNA sequence inserted into the lentiviral backbone plasmid pRRLsincppt.CMV.EGFP.wpre via MfeI sites (figure 1A).

### Construction of Cx36 expressing lentiviral vectors

The Cx36 coding sequence was excised from pcDNA3.2/V5/GW/D_rat Cx36 plasmid (GlaxoSmithKline) via SacI and AgeI restriction sites and sub cloned into the pRRLsinppt CMV.IRES.EGFP plasmid, between the CMV and IRES regions using SacI and XmaI restriction sites. The Cx36.IRES.EGFP sequence was excised from the resulting plasmid using SpeI and NotI restriction sites and the EGFP sequence was removed from the lentiviral backbone plasmid using XbaI and SalI restriction sites. The resulting plasmid construct is illustrated in figure 1A. Lentiviral vectors were prepared as previously described [16].

### Animals and primary culture

All animals were treated ethically in accordance with the rules of Bristol University.

Time-mated E18 and E14 rats were obtained from Harlan. Animals were terminally anaesthetized and embryos were removed from the uterus, then transferred into dissecting medium (Hank's balanced salt solution [HBSS, Gibco]).

### Neuronal cultures

E18 rat hippocampus was removed and dissociated by incubation in 10% [v/v] trypsin-EDTA/growth media for approximately 15 minutes followed by trituration to obtain a single cell suspension. Cells were plated in poly-D-lysine (0.1 mg/ml; Sigma) coated 24 well plates (7.5×10^4^ cells/well) in 500 µl growth media (Neurobasal medium [Gibco] supplemented with B27 [2%, Gibco] and penicillin/streptomycin [250U]). Cells were fed every 2–3 days and transductions were done at 3 days *in vitro* (DIV). Cells were maintained for a further 7 DIV when harvested for Western blot analysis. Striatal neurons were also grown in media containing DMEM∶Ham's F12 (2∶1); B27 (2%); penicillin/streptomycin (250 U); foetal calf serum (1% [v/v]) and were fed every 2–3 days.

### Striatal neurosphere culture

The striatum was removed at E14 from the embryos in HBSS and incubated for 10 minutes in accutase at 37 °C. After trituration, cells were seeded in polyhema (120 mg/ml, Sigma) coated 96 well plates at a density of 1×10^5^ cells per well in 100 µl media (DMEM∶Ham's F12 [3∶1] Gibco; B27 (2%); penicillin/streptomycin (250U); heparin (5 µg/ml); FGF2 (20 ng/ml); EGF (20 ng/ml). Transductions were performed whilst cells were in single cell suspension at a multiplicity of infection (MOI) of 5. Media was doubled the following day and at 3–4 days, neurospheres were transferred to a T25 flask where media added resulted in a five times dilution of the cells. Neurospheres were passaged every 7 days by re-seeding single cells at a density of 100 K/ml. At each passage samples were collected for RNA analysis by snap freezing in liquid nitrogen and storing at −80 °C and for differentiation studies. Briefly, cells were treated with accutase and triturated to obtain a single cell suspension and were spotted on poly-D-lysine coated coverslips in 24 well plates at a density of 5×10^4^ cells in 30 µl differentiation medium (DMEM∶Ham's F12 [3∶1]; B27 (2%); penicillin/streptomycin (250U) per well. Cells were maintained for 7 days before fixing in 4% paraformaldehyde.

### Western Blot Analysis

Transduced E18 primary neurons were washed once with PBS then incubated in RIPA lysis buffer (50 mM Tris-HCL pH 7.4; 150 mM NaCl; 1% Triton X-100; 1% sodium deoxycholate; 0.1% sodium dodecyl sulphate supplemented with protease inhibitors (Complete Mini, Roche)). Cells were harvested and homogenized with a needle and syringe prior to incubation on ice (20 minutes). The lysates were then centrifuged (>13,000×g, 20 minutes) and supernatants retained. Protein concentration was determined using the BCA protein assay (Pierce). Membranes were incubated with polyclonal rabbit anti-Cx36 (1∶500; Zymed 51-6300) or monoclonal mouse anti-alpha tubulin (1∶1000; Sigma) as the loading control. Horseradish peroxidase conjugated secondary antibodies were used (1∶10000; Amersham) and bound antibody was detected using ECL plus Western Blotting Detection Reagent (Amersham). Autoradiograph film (Amersham) was exposed to the membrane and developed using a Kodak film processor.

### Fluorescence Recovery After Photobleaching (FRAP)

E18 primary striatal neurons were plated in 35 mm glass-bottomed culture dishes (Iwaki) at a density of 4×10^5^ cells per dish in 2 ml media. Cells were transduced with either Cx36 shRNA or scrambled control lentiviruses (MOI 5) at 3 DIV and experiments performed 7 days post-transduction. Media was supplemented with 25 mM Hepes (Sigma) prior to performing FRAP experiments. Cells were incubated in the presence of 5-(6)-carboxyfluoresceine-diacetate (10 µM, CFDA; Invitrogen) diluted in HBSS for 5 minutes at room temperature followed by washing 3 times with HBSS and returned to their original media prior to FRAP measurements. Briefly, experiments were performed on a confocal microscope using the Leica SP2 system. Images were obtained using a 40× oil objective with zoom. A single cell within a cluster of cells was selected for bleaching for 5 frames for a total of 30 seconds and recovery recorded at 20 second intervals for a total of 15 minutes (45 frames). Frame sequences from each experiment were analyzed in ImageJ and the fluorescence intensity (F_i_) of each bleached cell and its subsequent recovery was analyzed. A non-bleached cell was also analyzed in order to correct for a loss of fluorescence during the acquisition process. The following formulae were used to calculate fluorescence level [17]: F_i_ of bleached cell = (F_t_−F_0_)/(F_pre_−F_0_), whereby Ft =  fluorescence at each recovery time point, F_0_ =  post-bleach intensity and F_pre_ =  pre-bleach fluorescence. To correct for bleaching during image acquisition: F_Norm(i)_ = Ft/Fpre, whereby FNorm(i) = F_i_ of unbleached cell. The final corrected value is calculated by F_i_/F_Norm(i)_. Data was analysed by repeated measures ANOVA and Bonferroni post-hoc test.

### Reverse Transcription and Polymerase Chain Reaction

RNA was isolated from frozen neurospheres using Trizol. Isolated RNA was DNaseI treated (Roche) and the preparation cleaned up using the RNeasy minelute kit (Qiagen). RNA quality and quantity was determined using spectrophotometry and RNA was reverse transcribed into cDNA using the Superscript III First Strand Synthesis Kit (Invitrogen) according to manufacturer's instructions. PCR primers for Cx36 and GAPDH were used as previously described [18]; [19]. PCR reactions were performed following the manufactures instructions.

### BrdU incorporation and Immunocytochemistry

Cells were pulsed with 5-bromo-2′-deoxyuridine (BrdU; 0.2 µM, Sigma) for 24 hours prior to differentiation. Antigen retrieval was performed following fixation of cells with 4% paraformaldehyde. Cells were incubated on ice in HCL (1 M, 10 mins) followed by HCl at 37 °C (2 M, 20 mins). The cells were then washed in borate buffer (0.1 M, 12 mins at room temperature) and incubated with sheep anti-BrdU (Abcam; 1∶500) overnight. Alexafluor secondary antibodies were used at 1∶500.

Immunostaining using O4 was performed on live cells. Briefly, cells were incubated with anti O4 (diluted 1∶4, derived from hybridoma cell lines, ECACC, Cambridge, UK) for 30 minutes at 37 °C. Cells were washed 3 times with DMEM before fixing. Cells were blocked and incubated with secondary anybodies as normal. For the remaining immunostaining, cells were fixed in 4% PFA and rinsed with phosphate buffered saline (PBS) before blocking and incubating with primary antibodies overnight at 4 °C. Primary antibodies and dilutions used were mouse monoclonal anti-βIII tubulin (1∶500, Covance), rabbit polyclonal anti-glial fibrillary acidic protein [GFAP] (1∶500, DAKO) and rabbit polyclonal anti-Cx36 (1∶400, Zymed). Cells were washed with PBS/Triton-X (0.1%) and incubated in the appropriate Alexafluor secondary antibodies (1∶500, Invitrogen). Coverslips were mounted using Fluorsave reagent (Calbiochem). Cell counts were performed on at least 3 fields per coverslip with at least 3 coverslips per treatment group 4 independent experiments were performed for each treatment group. All counts are expressed as a percentage of total cells. Cell body area, neurite branching and length of longest neurite were analyzed using the ImageJ plug-in NeuronJ. Statistical analysis was performed by one-way ANOVA followed by a Bonferroni post-hoc test.

## Results

### Lentiviral vectors mediate the effective overexpression and knockdown of Cx36

**Figure 2:**
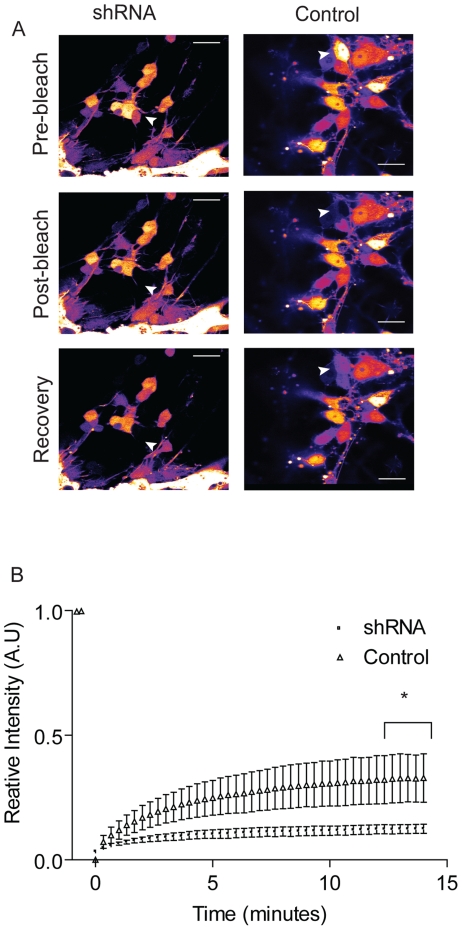
Cx36 shRNA- knockdown of gap junction intercellular communication. GJIC was assessed by fluorescence recovery after photobleaching in shRNA-transduced striatal neurons loaded with 6-(5) carboxyfluorescein diacetate. Arrow marks cell of interest (A). Pooled data showing relative fluorescence intensity from 3 separate cultures is shown (B). Scale bar = 20 µm. Error bars represent standard error of the mean.

To test the efficacy of Cx36 knockdown we initially used primary hippocampal neuronal cultures transduced with vectors encoding Cx36 or Cx36 shRNA (Figure 1A). Transduction levels of these cells were highly efficient, reaching levels of ∼100%. Cx36 protein levels were analysed by western blotting and the Cx36 shRNA was found to mediate highly effective knock down (Figure 1B) while the Cx36 vector increased expression in a concentration dependent manner (Figure 1C). To validate Cx36 knockdown a functional FRAP model was used to monitor gap junctional intracellular communication (GJIC). To achieve this Cx36 shRNA transduced primary neuronal cultures were loaded with the GJ-permeable dye CFDA and this was subsequently photobleached in a single cell. Transfer of dye from cell to cell occurs via GJs and this can be monitored by real time by microscopy. It was found that knockdown of Cx36 reduced dye recovery in photobleached cells whereas recovery was unaltered in control cells (Figure 2A, Movie S1and Movie S2). Cellular fluorescence intensity was pooled together and quantified (Figure 2B) and a statistically significant difference between recovery of fluorescence in Cx36 shRNA and control cells was observed (p<0.0001; Repeated measures 2-way ANOVA and Bonferroni post-hoc test).

**Figure 3:**
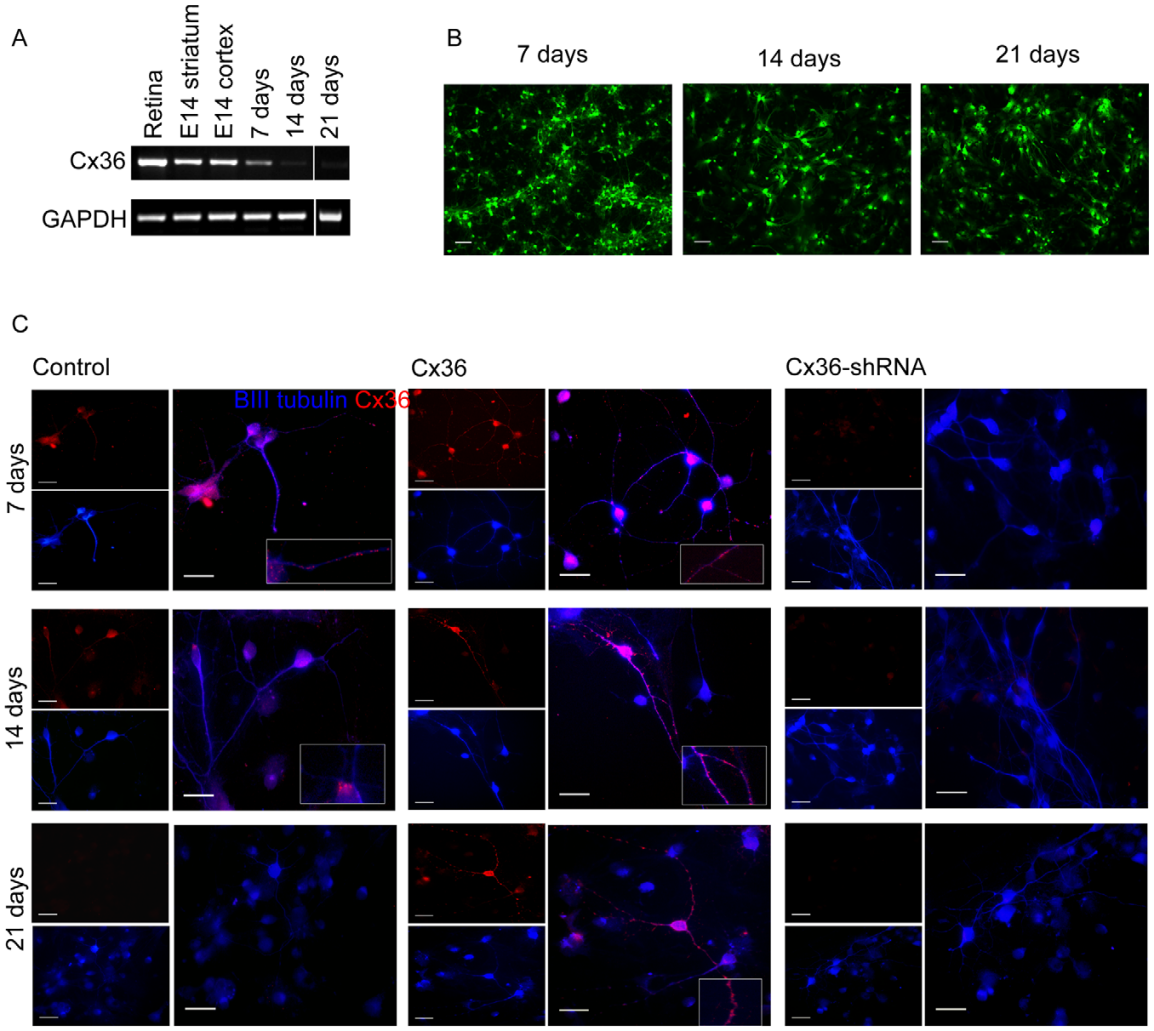
Expression of Cx36 in neurosphere cultures. Cx36 mRNA expression progressively decreases in neurosphere cultures as time *in vitro* increases (A). The lenti-Cx36-EGFP vector sustains expression throughout the culture period. Scale bar = 40 µm (B). Cx36 Immunostaining of differentiated neural cells following viral transduction (C). Scale bar = 20 µm.

During mammalian development, Cx36 is expressed in the striatum and in the mature brain Cx-36 mediated neuronal coupling remains. We therefore chose to study Cx-36 function in an E14 striatal neurosphere model and showed that our vectors mediated highly effective transduction, as assessed by EGFP expression (Figure 1D). Neurospheres formed after 3–4 days post-dissection all expressed EGFP. This expression persisted for up to 21 days in neurosphere culture and following differentiation, EGFP expression was maintained in ∼100% of cells (Figure 3B).

### Expression patterns of Cx36 in neurosphere cultures

Striatal NPCs were maintained as neurospheres and passaged every 7 days in order to allow expansion of progenitors. The effect of this on Cx36 expression was examined by RT-PCR. Levels of Cx36 mRNA were initially high in primary E14 striatal and cortical tissue. The maintenance of neurospheres in culture resulted in reduced Cx36 expression when measured every 7 days and it became undetectable by 21 DIV (Figure 3A). Transduction of these cells with a lentivirus expressing Cx36-EGFP or an anti-Cx36 shRNA followed by differentiation resulted in an increase or reduction in Cx36 mRNA levels respectively (as assessed by immunocytochemistry). EGFP expression was observed by auto fluorescence in differentiated cells from cultures up to 21 DIV (Figure 3B), indicating that the vector had successfully integrated into the genome of the host cell and was still expressing the coding cassette. Double staining for Cx36 and βIII tubulin revealed sustained over expression of Cx36 protein in neurons up to 21 DIV (Figure 3C) although this was found in fewer cells than at 7DIV. At 7 DIV Cx36 expression is mostly located within the cell body and, as time in culture increases, Cx36 assemblies had been trafficked to the dendrites as well. Boxed areas of figure 3C illustrate the dendritic location of Cx36.

### Cx36 regulates neuronal differentiation

**Figure 4:**
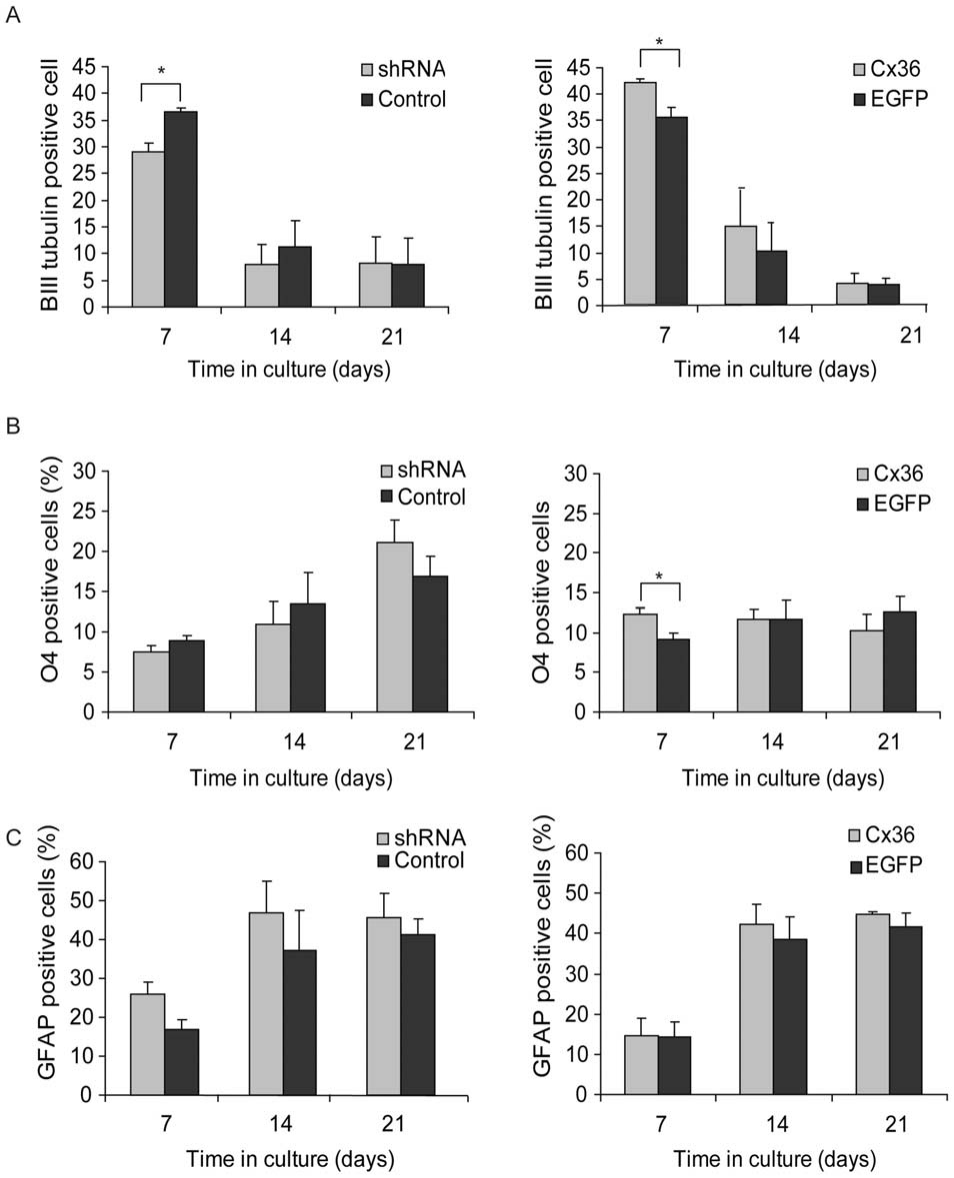
Cx36 influences cell fate. The lentiviral mediated expression of Cx36 increased neuronal differentiation, whereas shRNA mediated Cx36 knockdown decreased neuronal differentiation (A). Cx36 increased oligodendrocytes number whereas Cx 36 shRNA transduction has no effect on differentiation (B). No effect on astrocyte differentiation was observed (C). Maintenance of spheres in culture for longer periods had no effect on neural differerentiation. **p≤0.01; ***p≤0.001, one-way ANOVA with Bonferroni post-hoc test. Error represents standard error of the mean.

**Figure 5:**
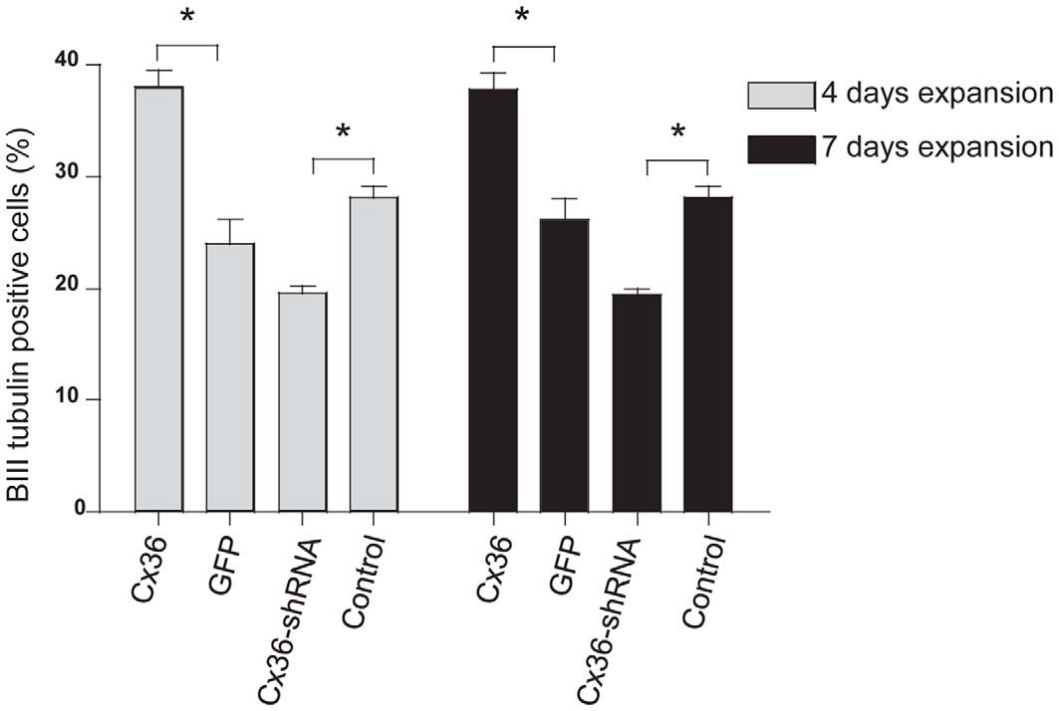
Cx36 influences cell fate after 4 and 7 days expansion. Cx36 expression increased neuronal differentiation following 4 and 7 days expansion, whereas Cx36 knockdown decreased it. *P<0.05 versus relevant control (by one-way ANOVA with Bonferroni post-hoc test). Control for Cx36 is GFP and control for Cx36-shRNA is scrambled control labelled as control in the figure.

**Figure 6:**
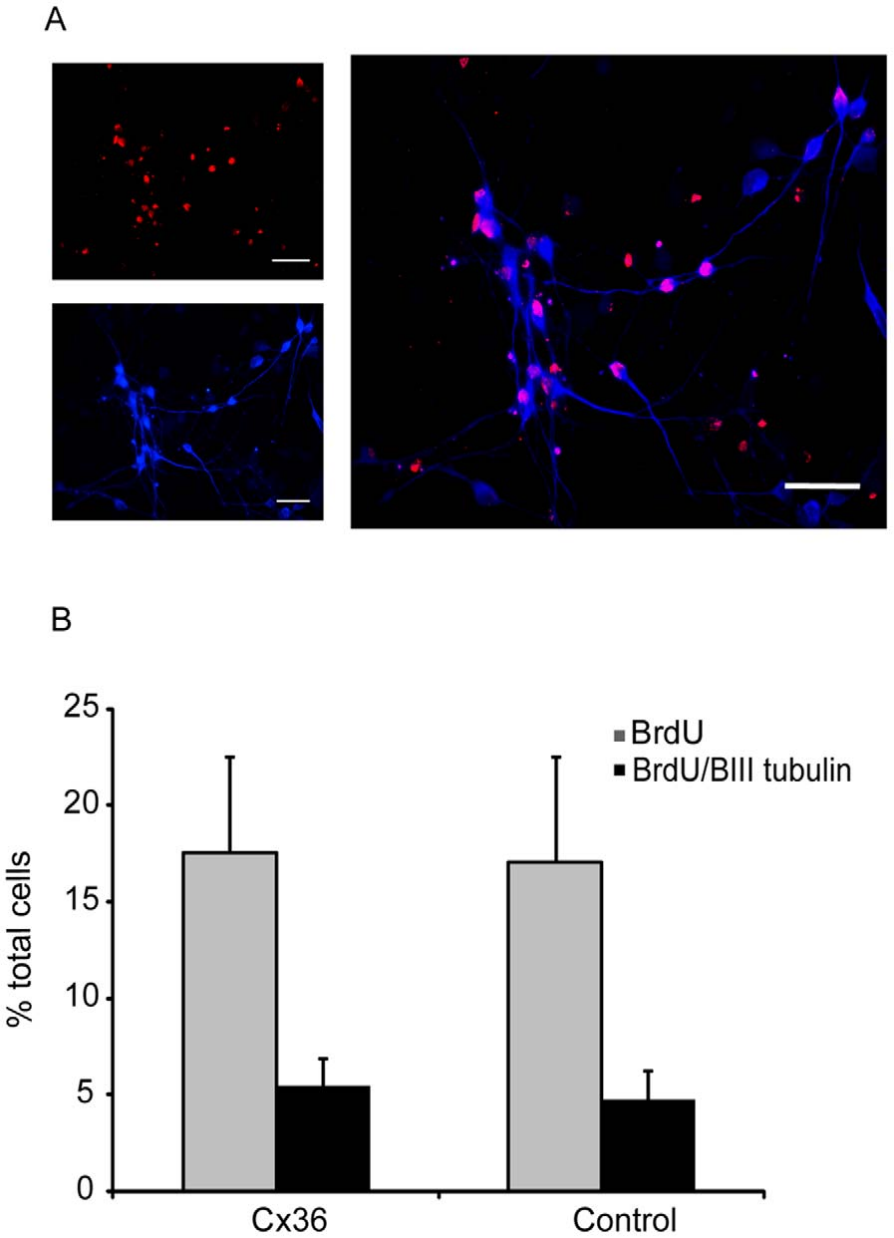
Analysis of cell proliferation in neuronal precursor cultures. Double-labelling for BIII tubulin and BrdU (A). Scale bar 40 um. There was no significant difference in proliferation of neuronal precursor cells (BIII tubulin/BrdU^+^) when Cx36 is over expressed compared with control (B). Error bars represent standard error of the mean.

The shRNA mediated knockdown of Cx36 resulted in a significant decrease (p = 0.0001) in the number of βIII tubulin positive cells (Figure 4) differentiating 7 days post-transduction (29.05±1.65%) compared to cells transduced with the scrambled control (36.63±0.63%). The data also show there is no change in cell counts at the 14 DIV or 21 DIV time points. In parallel with this data, over expression of Cx36 resulted in a significant increase (p<0.01) in the number of βIII tubulin positive cells (42.13±0.71%) compared to the EGFP control (35.65±1.77%). Again there were no significant changes at 14 and 21 day time points (Figure 4A). However, oligodendrocyte differentiation was found to be altered following Cx36 overexpression. A significant increase (p = 0.0001, one-way ANOVA with Bonferroni post-hoc test) in the number of O4 positive cells observed at 7 DIV following Cx36 over expression (Cx36: 12.31±0.84% and EGFP control: 9.20±0.75%). Cx36 shRNA treated cells also show a modest decrease in the number of O4 positive cells yet this did reach statistical significance (Figure 4B). Finally, the number of GFAP positive cells was analyzed and no significant changes were observed in cells transduced with the Cx36 lentivirus compared with the control (figure 4C). Knockdown of Cx36 produced a significant increase in the number of GFAP positive cells (p<0.01; 26.07±2.87%) compared with the control group (17.3±2.27%) at the 7DIV time point. Over 95% of neurons that spontaneously differentiated were GABA positive (Figure S1). To observe any early differential changes (prior to 7DIV) caused by viral transduction βIII tubulin counts were carried out after 4 (and 7) days expansion (Figure 5). The results showed that βIII tubulin counts were increased significantly at 4 (and again at 7) days following Cx36 transduction and were decreased significantly following transduction with the Cx36 shRNA (Figure 5). Furthermore, there was no change in trypan blue (total) counts after 4 or 7 expansion (data not shown). The proliferation of neuronal precursors at 7 DIV was assessed by BrdU incorporation (Figure 6). This indicted the proliferation profile of precursors prior to differentiation and, whilst a small number of cells will continue to proliferate following growth factor removal, the comparison between proliferation of neuronal precursors in Cx36 transduced and control cells was analysed. Co-localization of the neuronal marker βIII tubulin and BrdU did not significantly differ between cells transduced with Cx36 (5.39±1.53%) and the control (4.67±1.56%) (Student's T-test) suggesting that there was no difference between the proliferating neuronal precursors.

**Figure 7:**
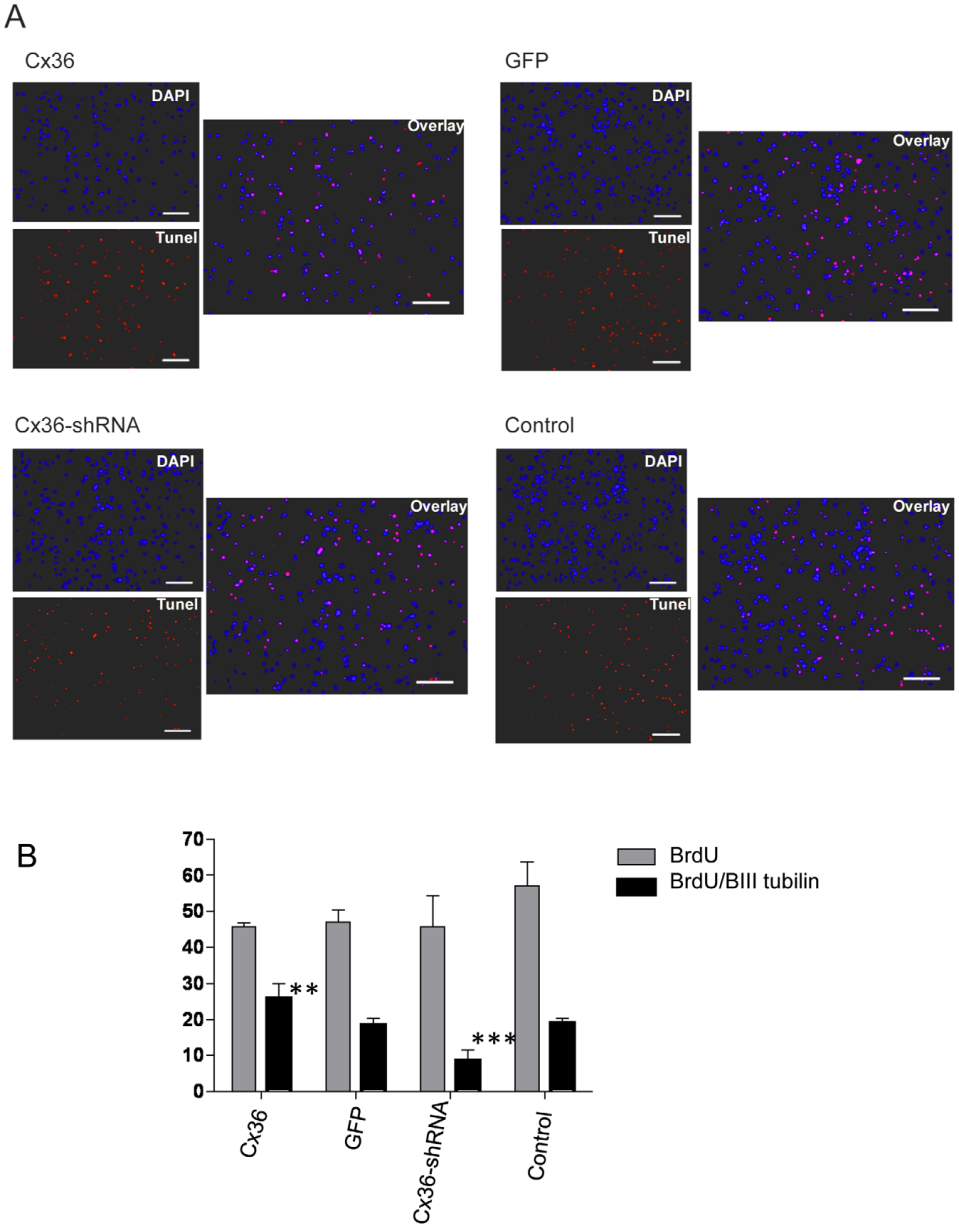
Tunel assay. Tunel+ nuclei (red) of neural precursor cells following 4 days expansion. Dapi (blue). Scale bar = 100 um. (A). Percentages of Tunel positive cells showed no significant difference in the number of apoptotic cells within the four different conditions at 4 and 7 days expansion (B).

To ensure any early differential changes (prior to 7DIV) in cell viability/proliferation were observed we counted apoptotic cells using Tunel staining (Figure 7) and again measured BrdU incorporation after 4 and 7 days expansion (Figure S1). The results showed there was no change in the number of apoptotic (Tunel stained) cells after 4 or 7 days expansion when control cells were compared with shCx36 or Cx36 transduced cells (Figure 7). Furthermore, a similar comparison showed was there was no change in BrdU incorporation in total cells or in neurons (Figure S1) after 4 (or 7) days. Statistical analyses of the results showed that βIII tubulin counts were increased significantly at 4 (and 7) days following Cx36 transduction and were decreased significantly following transduction with the Cx36 shRNA (Figure 5 and Figure S1). These data together with the βIII tubulin counts show that viral-mediated manipulation of Cx36 in neurosphere cultures had no effect on cell growth kinetics and cultures continued to expand at a similar rate in all conditions.

No significant changes were observed in cell body area or length or longest neurite in any of the treatment conditions. Though, overexpression of Cx36 caused a significant increase in the total number of neurites (one way ANOVA p<0.05) at 14 DIV, (5.55±0.42) compared to the control (4.86±0.16). At all other time points and in the Cx36 shRNA-treated cells there was no difference in neurite branching (Figure S2). In order to investigate whether Cx36 had the ability to alter neuronal fate in established cultures, cells that had already been maintained for 7 days were transduced with Cx36. The Cx36-mediated increase in neuronal differentiation was only observed when cells were transduced at early time-points as there was no change in the number of differentiating neurons when cells were transduced following 7 days in culture, when endogenous Cx36 levels had declined, suggesting that the cultures had lost the ability to respond to Cx36 over expression (Figure S3).

## Discussion

In the developing brain, neurons and their positions are established and then glial and other cells are matched to the architecture of the network [20]. Foetal neurospheres maintained in culture contain a mixture of multipotent and lineage restricted cell types that can be differentiated to give rise to neurons, astrocytes and oligodendrocytes [21], [22]. Differentiation in the brain and in neurosphere models is known to be governed via intrinsic pathways and via responses to extracellular cues and by cell-cell contact [23], [24], all of which can be regulated by Cxs. However, Cx36 is the only Cx identified to be expressed in neurons [7], [25]. In this study we showed that shRNA vectors significantly reduce Cx36 protein expression and also inhibit GJIC. Additionally, altering Cx36 expression does not affect the growth of neurosphere cultures. Cx36 knock down resulted in significantly reduced neuronal differentiation while the overexpression of Cx36 increased neurogenesis. Furthermore, BrdU and Tunel experiments showed that the results were not a reflection of increased neuroblast proliferation prior to differentiation or differential apoptosis respectively as there were no differences between any of the experimental groups. The increased expression of Cx36 during development may provide enhanced cell-cell contact between neuronal progenitors and hence promote neuronal differentiation. A possibility supported by the dependence on Cx36 GJIC for neuronal coupling (also observed in this study). Interestingly, the knockdown of Cx36 resulted in a decrease in the number of neurons and an increase in the number of GFAP-positive cells, which could suggest the decrease in neuronal differentiation, elicits a compensatory rise in astrocyte differentiation. The effects of Cx36 manipulation on neurogenesis were only observed in cells that had been expanded for 7 days or less suggesting that the cells have already become committed and no longer respond to Cx36 over expression or knock down. This suggestion fits with the observed decrease in endogenous Cx36 expression that occurs in NPCs after 14–21 days and with Cx36 transduction of 7 day old cultures having no effect on neuronal differentiation. To explore further the involvement of Cx36-dependent gap junction communication in intra-neuronal communication and development experiments reflecting the in vivo composition of cells could be undertaken. This could be achieved using conditional Cx-36 transgenic mice and/or the stereotactic injection of viruses. Significantly following the lentiviral mediated overexpression of Cx36 in the intact hippocampus CA3 region of adult rats increased gamma oscillatory activity was measured (unpublished). Results which further support Cx36 playing a significant physiological role in intra-neuronal gap junction communication in intact networks.

In addition to the increase in the number of neurons following Cx36 over expression, we found the number of oligodendrocytes was also significantly increased. Oligodendrocytes are the myelinating cells of the CNS and act to insulate neuronal axons transmitting electrical impulses. It is also known that oligodendrocytes provide trophic factors that promote neuronal survival and hence there may be a cooperative relationship between neurons and oligodendrocytes during the differentiation process [26]. The rise in oligodendrocytes observed may hence be due increased need for myelination and trophic factor support. Studies on transgenic mice lacking both Olig genes revealed that differentiation of motorneurons and oligodendrocytes was replaced with differentiation of interneurons and astrocytes [27], [28]. It may also be the case that an increase in the number of neurons provides a more supportive environment for the differentiation of oligodendrocytes and may explain the changes observed.

Gap junctions have primarily been studied as an intracellular channel that connects one cell to another yet they have recently been described as having a number of other functions. Cxs have been shown to be necessary for radial glial migration in the developing neocortex by acting as dynamic anchoring points for the migrating cells [29]. Indeed, neuronal differentiation from mouse and human stem cells was induced by cell-cell contact and cells deficient in Cx43 show aberrant specification [30], [31]. Cxs may also function in embryonic development to coordinate coupled populations and as an adhesive contact between migrating cells [14]. Extensive coupling has been identified within hESC colonies [13], [32] and several other reports demonstrate that GJIC is required to maintain NPC populations in a proliferative state [33], [34]. During neuronal differentiation of NPCs, the expression profile of Cxs is dynamic [35]. Progenitor cell populations respond to growth factors in the culture media and Cxs themselves can be regulated by them [36]. Alterations in Cx36 expression may change the way in which NPCs respond to their extracellular environment leading to transformed neural fate decisions. The identification of hemichannels has also implicated Cxs in paracrine signalling. Purinergic signalling plays a major role in neural development, particularly during later developmental stages. Spontaneous radial glial Ca^2+^ waves have been recorded in the proliferative cortical ventricular zone (VZ). These waves are initiated by Cx hemichannels and disruption of this signalling reduces VZ proliferation [37]. This may be important in the activation of intracellular signalling pathways and upregulation of neural genes. Expression of Cx36 may allow the assembly of greater numbers of hemichannels and offer a greater reactive potential to purinergic signals and thereby allow a neuronal fate to be determined.

### Conclusions

Gap junctions are highly expressed in stem cell populations and they play important roles in mediating intra- and inter-cellular communication. However, the role that specific connexins play in cell specification has not been investigated. Here we report that lentiviral mediated Cx36 expression in NPC cultures mediated an increase in neuronal and oligodendrocyte differentiation. Importantly, knockdown of Cx36 significantly reduced the number of differentiated neurons and increased the number of differentiated astrocytes, suggesting an increase in glial progenitor proliferation following a failure in neuronal differentiation. The effects that we observed occurred shortly after NPC expansion suggesting that Cx36 may regulate early neurogenesis during development, perhaps to fine tune the establishment of neural networks within the brain.

## Supporting Information

Figure S1 A. GABAergic neurons Double staining for BIII tubulin (red) and GABA (green). Neurons that spontaneously differentiated were primarily GABAergic. Scale bar 40 um. B. Analysis of cell proliferation in neuronal precursor cultures following 4 days expansion. Neural precursor cells expanded for 4 days in the presence of BrdU showed no significant difference in BrdU incorporation in total cells or in neurons (BrdU+/BIII Tubulin+) when Cx36 was over expressed or knocked down compared with respective controls (A). Control for Cx36 is GFP and control for CX36-shRNA is scrambled control labeled as control in the figure. The results were analyzed by ANOVA followed by post-hoc Newman-Keuls multiple comparison test. There was no difference between any of the BrdU treatment groups. When the BrdU+/BIII Tubulin+results were compared: **P<0.01 when Cx36 treated cells were compared to its GFP controls; ***P<0.001 when Cx36-shRNA treated cells were compared to the scrambled controls. These results reflect the increase in neuronal BIII Tubulin+cells in the Cx36 treated group and decrease in BIII Tubulin+ cells in the Cx36-shRNA treated group. C. Analysis of cell proliferation in neural precursor cultures when BrdU was administered during the 7 day differentiation phase. Neural precursor cells expanded for 7 days in the absence of BrdU but differentiated for 7 days in the presence of BrdU showed no significant difference in proliferation rate when Cx36 was over expressed or knocked down compared with respective controls (A). Control for Cx36 is GFP and control for CX36-shRNA is scrambled control labeled as control in the figure.
(1.46 MB DOC)

Figure S2 Neurite analysis. The number of neurites was analyzed in Cx36 shRNA versus scrambled control treated cells (A) and also in Cx36 versus EGFP control treated cells (B). Primary, secondary, and tertiary branching was analyzed along with total neurites. *p = ≤0.05, Student's T Test.
(1.05 MB EPS)

Figure S3 Effect of Cx36 on differentiation of older NPC cultures. Neurospheres were transduced with Cx36 when their endogenous Cx36 mRNA levels had declined. There was no significant difference in the percentage of cells differentiating into neurons in Cx36-transduced cells compared to the control. Error bars represent standard error of the mean.
(0.43 MB EPS)

Movie S1 Frap experiment: Bleach in the presence of anti-Cx36-shRNA
(1.09 MB MP4)

Movie S2 Control FRAP experiment.
(1.10 MB MP4)
