# Supplementary figures and images for: Connexin 36 Expression Regulates Neuronal Differentiation from Neural Progenitor Cells
Source: PLoS One. 2011 Mar 9;6(3):e14746. doi: 10.1371/journal.pone.0014746 (PMC3052311; doi:10.1371/journal.pone.0014746)

A

B

**
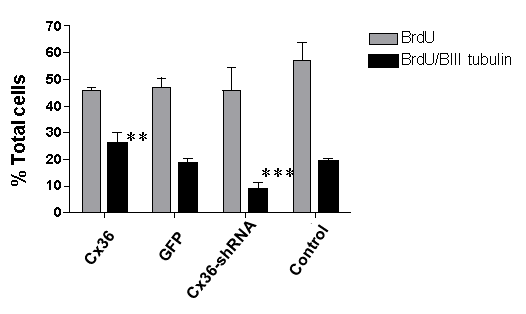
**

C

Supplement: Figure S1 — A. GABAergic neurons Double staining for BIII tubulin (red) and GABA (green). Neurons that spontaneously differentiated were primarily GABAergic. Scale bar 40 um. B. Analysis of cell proliferation in neuronal precursor cultures following 4 days expansion. Neural precursor cells expanded for 4 days in the presence of BrdU showed no significant difference in BrdU incorporation in total cells or in neurons (BrdU+/BIII Tubulin+) when Cx36 was over expressed or knocked down compared with respective controls (A). Control for Cx36 is GFP and control for CX36-shRNA is scrambled control labeled as control in the figure. The results were analyzed by ANOVA followed by post-hoc Newman-Keuls multiple comparison test. There was no difference between any of the BrdU treatment groups. When the BrdU+/BIII Tubulin+results were compared: **P<0.01 when Cx36 treated cells were compared to its GFP controls; ***P<0.001 when Cx36-shRNA treated cells were compared to the scrambled controls. These results reflect the increase in neuronal BIII Tubulin+cells in the Cx36 treated group and decrease in BIII Tubulin+ cells in the Cx36-shRNA treated group. C. Analysis of cell proliferation in neural precursor cultures when BrdU was administered during the 7 day differentiation phase. Neural precursor cells expanded for 7 days in the absence of BrdU but differentiated for 7 days in the presence of BrdU showed no significant difference in proliferation rate when Cx36 was over expressed or knocked down compared with respective controls (A). Control for Cx36 is GFP and control for CX36-shRNA is scrambled control labeled as control in the figure. (1.46 MB DOC) [file pone.0014746.s001.doc]
